# A scattered volume emitter micropixel architecture for ultra efficient light extraction from DUV LEDs

**DOI:** 10.1038/s41598-024-64689-y

**Published:** 2024-06-19

**Authors:** Faris Azim Ahmad Fajri, Anjan Mukherjee, Suraj Naskar, Ahmad Fakhrurrazi Ahmad Noorden, Aimi Abass

**Affiliations:** 1 ams OSRAM Group, Leibnizstraße 2, Regensburg, Germany; 2 Centre for Advanced Optoelectronics Research, Kulliyyah of Science, International Islamic University Malaysia, Kuantan, Pahang Malaysia

**Keywords:** Materials science, Optics and photonics, Physics, Electrical and electronic engineering

## Abstract

Deep ultraviolet light-emitting diodes (DUV LEDs) typically suffer from strong parasitic absorption in the p-epitaxial layer and rear metal contact/mirror. This problem is exacerbated by a substantial portion of the multiple quantum well (MQW) emissions having a strong out-of-plane dipole component, contributing to emission in widely oblique directions outside the exit cone of the front semiconductor emitting surface. To address this, we propose an architecture that leverages such a heavily oblique angular emission profile by utilizing spaced-apart or scattered volume emitter micropixels that are embedded in a low-index dielectric buffer film with a patterned top surface. This approach achieves high light extraction efficiency at the expense of enlarging the effective emission area, however, it does not require a high-index (e.g., sapphire) substrate or a lens or a nanotextured epi for outcoupling purposes. Hybrid wave and ray optical simulations demonstrated a remarkable larger than three to sixfold increase in light extraction efficiency as compared to that of a conventional planar LED design with a sapphire substrate depending on the assumed epi layer absorption, pixel size, and ratio of light emission area to the MQW active area. An extraction efficiency three times greater than that of a recent nanotextured DUV LED design was also demonstrated. This architecture paves the way for DUV LEDs to have a plug efficiency comparable to that of mercury lamps while being significantly smaller.

## Introduction

Deep ultraviolet (UV) light-emitting diodes (LEDs) covering 100 nm to 400 nm wavelengths (UV-C and UV-B, respectively) have various applications in disinfection^1,2^, sterilization^3,4^, phototherapy^5,6^, plant growth^7,8^, water/food decontamination^9,10^, and sensing^11,12^. It has great potential to replace harmful mercury-based lamps as the primary UV light source because aluminum gallium nitride (AlGaN)-based emitters are environmentally friendly, cost effective, highly robust, and energy efficient^13–15^. Recent studies have suggested that skin problems associated with UVLEDs should be avoided in the future^16,17^. At present, their optical performance is quite weak with the best external quantum efficiency (EQE) being only approximately 20%^18^, while that of commercially available materials is only 10% WPE^19^. The light extraction efficiency (LEE) and internal quantum efficiency (IQE) are crucial for an excellent EQE, as a low LEE of approximately 20% is the best EQE limiting factor, compared to an IQE of approximately 60%.

The main challenges of DUV AlGaN emitters stem from a considerable contribution of transverse magnetic (TM) dipole emission in the multiple quantum wells (MQWs), which generate light that propagates at an opaque angle to the surface normal^20^; large total internal reflection (TIR) and Fresnel-related loss due to the large refractive index (RI) contrast between AlGaN and air^21^; and strong radiation-absorbing p-side epi and contact materials due to inefficient hole injection and a nonohmic contact formation layer^22^. Since the early 2000s, researchers have been working on improving the performance of DUV LEDs. Examples include improving the uniformity of current spreading by improving electrode placement and patterning^23,24^, encapsulating chips with extraction-enhancing materials^25–27^, growing epilayers based on densely packed nanorod or nanowire structures^28,29^, and applying highly reflective photonic crystals to epilayers^30–32^.

Furthermore, various methods in designing the chip have been studied, including improving the LEE by utilizing micro/nanopatterning on specific layers, where this patterning provides a high probability of surface extraction. Khizar et al. enhanced the output power by 55% using microlenses on a sapphire substrate^33^, Zhou et al. increased 2.5 times the light extraction by controlled roughening of the AlGaN layer’s nitrogen face^34^, Zhao et al. improved the LEE reaching 12% by patterning microdomes at the p-side^20^, Lee et al. presented a 67% output power enhancement by injecting air voids in a patterned sapphire substrate (PSS)^35^, Zheng et al. achieved an approximately 18% LEE by nanomeshing the p-contact^36^, and López-Fraguas et al. tripled the LEE, also reaching 12% by nanostructuring the p-contact metal^37^.

**Figure 1:**
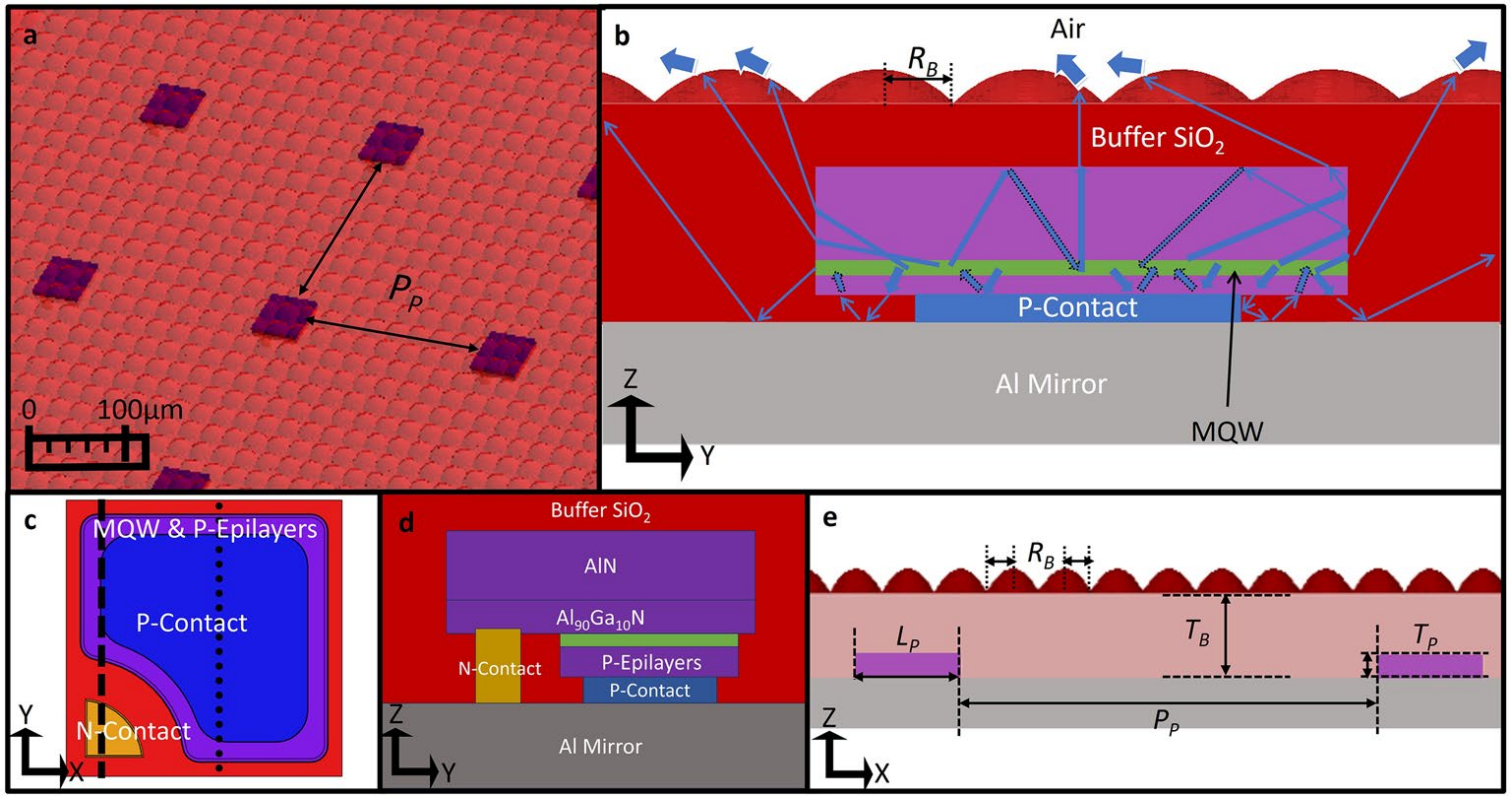
The scattered volume-emitter pixel (SVEP) architecture: The pixels are surrounded by the outcoupling structure, which is the silicon dioxide buffer layer at the top and the aluminum mirror at the bottom. (**a**) Is a sketch of an SVEP chip realization with a square lattice placement. (**b**) Gives a YZ cross-section view through the center of a pixel along with sketches of how light rays would propagate through the structure to be outcoupled into the air (blue arrows). (**c**) Is the top view of the XY plane over a single pixel with the n-epilayers removed. (**b**) Is a YZ cross-section along the dotted line of (**c**) while (**d**) is a YZ cross-section along the dashed line in (**c**). (**e**) Summarizes the geometrical parameters of importance for the LEE which are further discussed in the results section. These parameters are the hemispherical buffer texture radius, ${R}_{B}$, the buffer thickness, ${T}_{B}$, the squarish pixel length, ${L}_{P}$, and the pixel thickness,* T*_*P*_.

In this paper, a novel scattered volume-emitter pixel (SVEP) architecture is proposed to significantly enhance the light extraction efficiency without an outcoupling texture or roughening introduced at the semiconductor epilayer surfaces. The architecture consists of volume-emitter pixels in the micron size range (micropixels) that are placed with a large enough spatial distance to each other on a minimally lossy reflective substrate (here we consider a substrate with an aluminum metal coating) and enclosed by a thin lossless (or with extremely low absorption) low-index buffer sheet with a textured top surface, as visualized in Fig. 1. With such an architecture, light emitted by multiple quantum wells MQWs has a high probability of leaving the semiconductor region that has strong parasitic absorption with minimal round trips provided that the pixels are small enough. Light that exits the semiconductor region subsequently enters a buffer region that has significantly less parasitic absorption and a lower RI. Due to the smaller RI and absorption losses in the buffer region, there is less complexity in facilitating the outcoupling of light from the buffer region to air as long as cross-coupling between the semiconductor pixels is minimized via an increased separation and/or a proper placement strategy. As we demonstrate in the next sections, one can utilize easy to fabricate large micron-sized textures at the buffer layer-to-air interface to achieve highly efficient outcoupling of light. The SVEP architecture thus allow one to achieve a very high LEE without an expensive nanoscale outcoupling texture, lens, or a high-index (super)substrate, although at the expense of having an extended light outcoupling area as compared to the active MQW area. DUV LEDs are typically fabricated on a sapphire substrate which is typically not removed for light extraction purposes due to its high refractive index. However, as our SVEP approach does not rely on such high index substrates, one can detach the semiconductor epitaxial layers from the sapphire substrate during fabrication to save costs, provided that the substrate can be reused again afterwards. Though there is no industrial standard established just yet for detaching the DUV LED’s semiconductor epitaxial layer from the growth sapphire substrate, it has been shown that in principle it is achievable through thinning^38^, laser lift-off^39–42^, or chemical etching^43,44^. Ideally, the epilayers would be transferred to an intermediate carrier substrate for further processing steps (eg. contact depositions and singulations). Once the volumetric emitting pixels are ready, they can again be transferred (with distance between pixels implemented) to a final substrate with metal contacts and an aluminum reflector.

To provide a proof of principle demonstration on how the SVEP architecture would perform, hybrid wave and ray optical simulations were conducted. Such a hybrid modeling approach is especially needed to show the main advantage of the approach that even with relatively large pixel and buffer outcoupling texture sizes as compared to the emission wavelength, one can achieve an extraction efficiency that is 6 times greater or even more than that of a typical large-area sapphire chip architecture as shown below. We consider an $n\times n$ matrix of square LED pixels enclosed by a silicon dioxide (SiO_2_) buffer layer and placed on an Al-coated substrate that is highly reflective for UV-C radiation. Figure 1 illustrates the SVEP architecture based on several perspectives. Figure 1a provides a magnified view of the SVEP architecture. The blue squares are the volume emitter micropixels. The SiO_2_ buffer region is considered to have a simple micron-sized hemisphere texture with a square lattice placement. The cross section of a single pixel is given in Fig. 1b–d, where 1b and d are cross sections cut from the dotted line and dashed line of 1c, respectively.

Figure 1b also provides a sketch (blue arrows) of how light rays propagate through our SVEP architecture. Light emitted by the MQWs escapes the lossy semiconductor region into the buffer layer mainly through the pixel sidewalls and is subsequently outcoupled into air after possibly bouncing in the buffer region. As shown in Fig. 1c, the n-contact of each pixel is placed at a corner, which thus minimally interacts with the light emitted by the MQW. The n-contact placement choice is not optimal but is sufficient for demonstrating the strength of the approach. Figure 1d provides a more resolved layer stack description. Unlike most other approaches, the proposed SVEP architecture does not need a high-index superstrate (sapphire) or a lens on top to achieve high outcoupling efficiency although one can certainly still have it without impacting the light extraction performance (however at a heavy economical cost). The geometrical parameters depicted in Fig. 1e are the ones considered in this paper as they significantly influence the light extraction performance.

As previously mentioned, the SVEP architecture essentially enhances the outcoupling by first allowing as much light as possible to escape into a less lossy buffer region, where coupling into the air is also readily achievable due to a smaller refractive index contrast. Naturally, one must prevent light from reentering semiconductor regions with heavy losses again (e.g., in neighboring pixels) as much as possible. However, for the SVEP architecture to be able to provide strong outcoupling enhancement, one must first ensure that sufficient light emitted by the MQW can enter the typically less lossy n-epilayers or the thick undoped side of the semiconductor (in this case, AlN) instead of being directly lost in the p-epi layers or the p-contact. For this to be the case, the active MQW region should preferably have an RI that is as similar as possible to the n-side to minimize waveguiding losses in the p-side and active regions. In addition, the p-epi layers should not have an overly large bulk absorption such that most of the light emitted by the MQW is directly absorbed via evanescent coupling of the dipole emission^45^.

Unfortunately, not all UV-C semiconductor epilayer stack designs would allow this. Various works by other authors have considered or proposed epilayer stacks for UVC LEDs with different refractive indices. Many of these studies consider a rather low refractive index of the active region, a low extinction coefficient for "the p-side" or both^46–49^. To show how the SVEP architecture performs for different active region refractive indices and p-side loss regimes on the performance of the SVEP architecture, the following epilayer stack cases are considered:

1. The epilayer stack, as considered by López-Fraguas et al.^37^, has a very lossy p-side and significantly greater (effective) RI in the MQW region and p-epi layers as compared to the n-epi layers. The strong RI contrast leads to waveguide modes in these lossy layers and thus a strong reduction of light reaching the n-side.
2. The same epi stack as (1) but with an MQW RI of 2.4775 follows UCSB^46^ and Liu et al.^50^. Thus, there are fewer waveguided modes in the MQW and p-side regions.
3. The same as epi stack 2 but additionally with p-side layers, as considered by Liu et al., which have lower extinction coefficients^50^. With lower loss, even more power reaches the AlN layer due to less evanescent coupling-mediated losses^45^.

We consider these epilayer stacks to be representative of what is currently available. Obviously, the internal quantum efficiency of each epi stack may be vastly different, but it is not always clear which one would be the best at providing the highest IQE, and that is not the subject of the current publication, which focuses only on light extraction efficiency. Each of these epi stack cases with different RIs is labeled as “Reference”, “Low MQW RI”, or “Low P-side Loss” epi stacks. Simulations to show the outcoupling performance of the SVEP architecture were performed for all three epi stack cases. The specific layer thicknesses and refractive indices of these three epi stack cases are recorded in Table 1.

**Table 1:** Epilayer thickness and optical properties.

Materials		Thickness	Refractive index and extinction coefficient		
			Reference	Low MQW RI	Low P-side loss
1	Buffer sheet	10 µm above the AlN	1.5075 + i0.0002 (SiO_2_)		
2	AlN	4 µm	2.3147 (AlN)		2.3480 (AlN)
3	N-epilayer	1 µm	2.3723 (Al_90_Ga_10_N)		2.4285 (Al_70_Ga_30_N)
4	MQWs	18 nm	2.9532 (Al_60_Ga_40_N)	2.4775 (Al_61_Ga_39_N)	
5	P-epilayer	25 nm	2.4774 + i0.1190 (Al_75_Ga_25_N)		2.378 + i0.0053 (Al_93.5_Ga_6.5_N)
6	P-epilayer	75 nm	2.6103 + i0.3400 (Al_28.5_Ga_71.5_N)		2.4285 + i0.0248 (Al_70_Ga_30_N)
7	P-epilayer	40 nm	2.6981 + i0.4760 (GaN)		2.6110 + i0.4760 (GaN)
9	P-contact	30 nm	1.1449 + i2.4307 (Pt)		
10	N-contact	200 nm	1.7000 + i2.4500 (V)		
11	Rear mirror	9 µm	0.2137 + i3.1494 (Al)		

## Results and discussion

In this section, the impact of the most relevant geometrical parameters that strongly influence the LEE and reveal the physical mechanism of the extraction enhancement are discussed. These parameters are the pixel side length (${L}_{P}$), pixel placement periodicity (${P}_{P}$), buffer texture radius (${R}_{B}$), and thickness of the AlN layers (${T}_{AlN}$). Some of the other parameters that only weakly impact the LEE, e.g., the buffer layer thickness and air particles, are discussed in the second section of Supplementary Material.

For comparison with current industrial UVC LED solutions, we provide the LEE for the case of a large-volume emitter sapphire LED chip (with LED layers placed on a sapphire substrate) with a 1 $\times$ 1 mm^2^ surface area and a sapphire thickness of 400 µm. Details of this large sapphire LED chip reference calculation can be found in the supporting information (Fig. S1). With such a large chip architecture, one obtains an LEE of 0.048 (4.8%) for the “Reference” epi stack case (larger than the 4.6% reported by López-Fraguas et al.’s semi-infinite planar calculations^37^ due to some light escaping through the sapphire sidewalls in our simulations). For the “Low MQW RI” and “Low P-side Loss” epi stack cases, the large chip architecture provides LEE values of 0.065 (6.5%) and 0.054 (5.4%), respectively. These large sapphire chip LEE values are depicted in all LEE figures as the three horizontal dotted lines at the bottom of each figure. Comparing the three epi cases, although the “Low P-side Loss” power ratio entering n-epi is the highest, its sapphire chip counterpart LEE is lower than the “Low MQW RI”. This is due to the "Low P-side Loss” epi stack case producing an angular emission profile which has a significantly low radiant intensity in the 0^∘^–60^∘^ angle range (with 0^∘^ top normal direction) and high intensity around the $\sim$ 80^∘^ angle (refer to Fig. 7 in the “Methodology” section); thus, the TIR related losses is enlarged in the planar large area sapphire architecture for the “Low P-side Loss” epi stack case.

To compare the obtained LEE with respect to the theoretical maximum value for each epi-stack case, we also provide the portion of light emitted by the MQW that initially enters the n-epi in the first pass, which is shown as the top horizontal dashed line in all the LEE figures for each epi stack case in each figure. These theoretical maximum LEE values are obtained from analytical wave-optical dipole emission calculations in a semi-infinite multilayer system (p-contact/p-epi/n-epi) for each of the epi stacks, as detailed further in the “Methodology” section^51^. The absorption loss in the p-epilayers and p contact for all 3 epi stack cases are so dominant that the portion of light that enters the n-epi in the first pass can be less than half of the total light emitted by the MQWs. The portion of light power that enters the n-epi region is 0.2964 (29.64%), 0.4361 (43.61%), and 0.5496 (54.96%) for the “Reference”, “Low MQW RI, and “low P-side Loss” epi cases, respectively. Essentially, they represent the maximum LEE one can obtain for a given epi stack case.

In the following figures, we demonstrate how the SVEP architecture achieves strong LEE enhancement for all three epi cases. For each geometrical variation, the higher the power ratio that enters the n-epilayers, the greater the LEE achievable by the SVEP architecture. This naturally means that by utilizing the SVEP design, the “Low P-side Loss” case exhibits the highest extraction efficiency in all geometric variations (even for the cases discussed in the Supplementary Materials, second section).

### Pixel side length and periodicity

**Figure 2:**
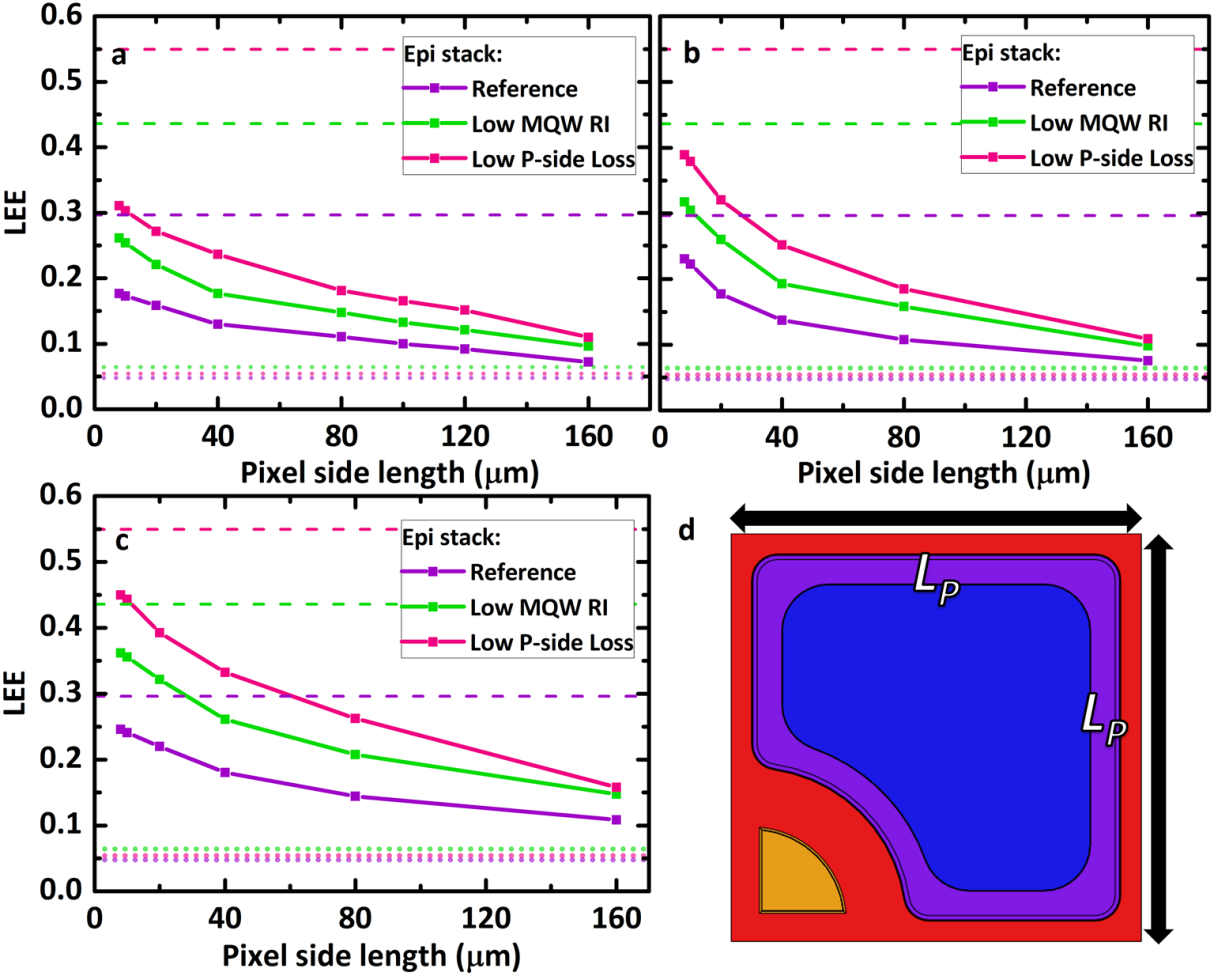
The LEE as a function of pixel size where (**a**) the pixel periodicity is 3 ${L}_{P}$, (**b**) the pixel periodicity is 100 ${L}_{P}$, and (**c**) when the ambient material is SiO_2_ (approximating an infinite buffer sheet). (**d**) depicts the single pixel side length, ${L}_{P}$. For this approach, the AlN thickness is 4 µm, buffer thickness and texture radius are 14 µm and 1 µm, respectively.

Figure 2 shows the LEE as a function of the pixel side length, ${L}_{P}$. (a) Pixel periodicity, ${P}_{P}=3{L}_{P}$, which corresponds to an emission area that is $\sim$ 9 times larger than the pixel surface area. This case represents a practical realization case in which one does not overextend the emission area with regards to the active area. (b) is the case when ${P}_{P}=100{L}_{P}$, which is the case where the pixels are practically very far apart from each other and minimal coupling into a neighboring pixel occurs. This case provides a picture of the maximum enhancement this architecture can provide. (c) is where light is extracted to an ambient infinite SiO_2_ media to show how much light exits the pixel and enters the SiO_2_ buffer layer without considering any back scattering or cross-coupling effect. The pixel shape remains the same through the variation of ${L}_{P}$, i.e., the design has the same thickness but varies in its lateral size (increasing scale). Here, we consider a buffer layer texture radius ${R}_{B}$ of 1 µm, thickness of 14 µm, and AlN thickness ${T}_{AlN}$ of 4 µm. (d) A sketch of the parameters that are being varied. We consider the range of ${L}_{P}$ only down to $8$ µm. For pixels less than 8 µm in length, arguably one would need a full wave-optic treatment of the UV-C pixel domain, which would be computationally expensive. Below this range, the ray optics approximation in our calculations breaks down. However, the increasing LEE trend that is visible in our calculations as we move to smaller pixels would most likely remain even for pixel sizes where the wave-optical effects dominate. This is because the main principle that light emitted by the MQWs would have a shorter distance to travel to the sidewall exit surfaces when the pixel size is smaller remains true.

As shown in Fig. 2a, we demonstrate a 3$\times$ LEE enhancement for a pixel side length of ${L}_{P}\sim 20$ µm and an almost quadruple improvement (4$\times$) at a pixel side length of ${L}_{P}=8$ µm without the need to introduce a nanophotonic outcoupling texture. Even at large ${L}_{P}=160$ µm, we show an LEE that is 1.5$\times$ greater than the planar reference of López-Fraguas et al. The LEE increases monotonically for all three epi stack cases as the UV-C pixel size decreases (${L}_{P}$ decreases). The “Low P-side Loss” epistack case shows a larger absolute change from 0.054 (5.4%) for the 1 $\times$ 1 mm^2^ large sapphire chip case to 0.31 (31% light outcoupled) at ${L}_{P}=8$ µm. The lowest change is for the “Reference” epi stack, from $0.075$ to 0.18. Even the LEE of the “Low MQW RI” epi stack case represents a middle road case where the LEE increases to 0.26 (at ${L}_{P}=8$ µm).

Figure 2b shows a similar trend but with a more drastic change in the increase in LEE as we move to smaller pixel sizes. This is because the pixels are thus far apart, and there is practically no cross-coupling of light between pixels. Our simulations show that LEEs of 0.24, 0.31, and 0.39 can be achieved at ${L}_{P}=8$ µm, which essentially shows that when one ensures that cross-coupling is minimal, one would obtain a far larger LEE enhancement, even reaching more than 5$\times$ of the planar reference 1 $\times$ 1 mm^2^ sapphire chip. There are other ways to minimize cross-coupling between pixels, e.g., zig-zag pixel placement or additional deflecting structures in the buffer region. Comparing the LEE values represented by the top dashed horizontal lines with the LEE values at ${L}_{P}=8$ µm, more than 70–80% of the light that passes through the n layers is extracted with the SVEP architecture when the pixel size is small enough and cross-coupling is avoided. Without any nanophotonic structuring, the architecture can operate close to the possible theoretical optimum. The different epi stack cases show differences in the relative percentage of light that enters the n-epi, which is ultimately outcoupled into the air. This is due to the difference in the angular distribution of the light that enters the n-Epi.

Figure 2c exhibits a similar picture to that of 2a and 2b, but the LEE numbers now reflect the percentage of power that reaches the SiO_2_ buffer region. Comparing the LEE values represented by the top dashed horizontal lines with the LEE line plots of the different epi stacks, we see that ~ 70–90% of the power that leaves the active region and enters the n-Epi can actually be extracted into the buffer with a µLED pixel size that is not overly small ${L}_{P}\sim <20$ µm. By comparing the absolute numbers between 2b) and c), one can see that approximately 10% of the light that reaches the buffer will be lost in the buffer due to imperfect outcoupling of light from the buffer into air (e.g., at ${L}_{P}=8$ µm LEE $\sim$ 0.45 in Fig. 2c as opposed to LEE $\sim$ 0.4 in b for the case of the “Low P-side Loss” epistack). This limitation is in part due to the use of a buffer texture that is unoptimized and only operates in the ray-optics regime per our approximation. However, most of the light that manages to escape into the buffer region can be directly outcoupled.

**Figure 3:**
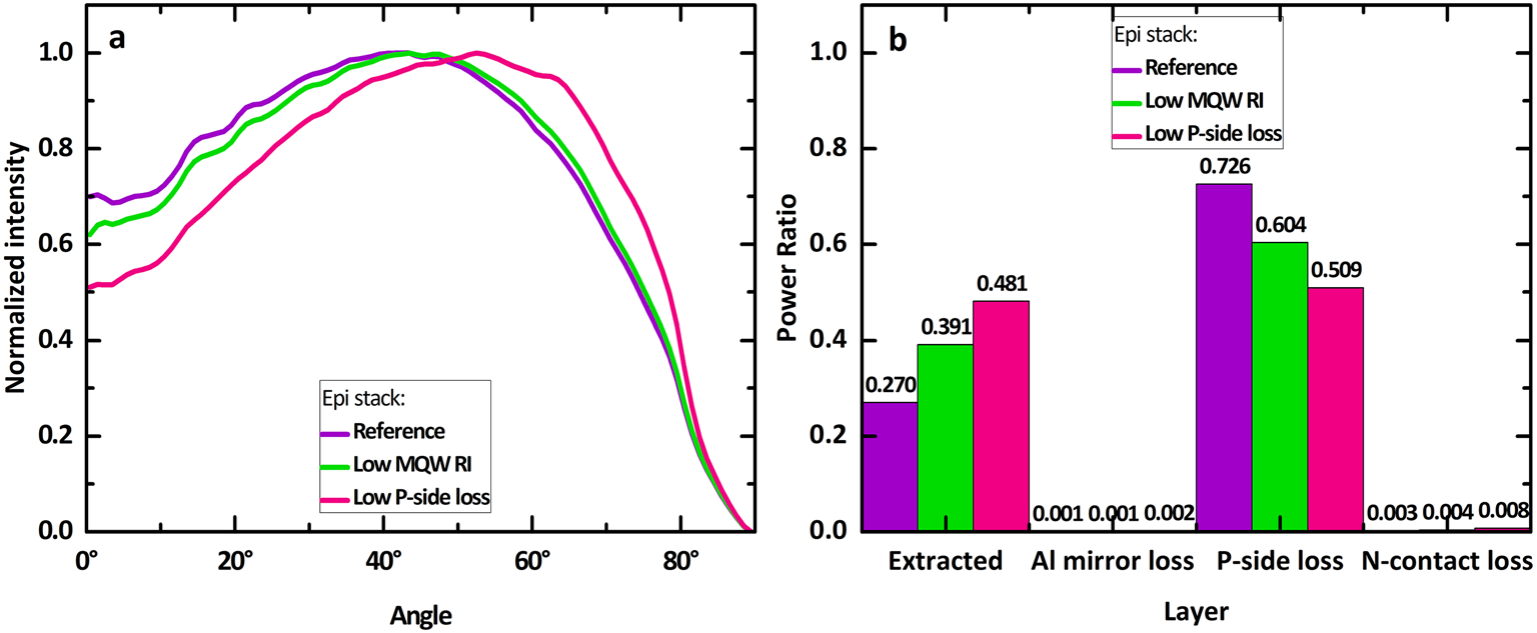
(**a**) is the light intensity as a function of the polar angle and (**b**) is a histogram chart summarizing the extraction and losses for three epi stack cases. In (**a**), ${L}_{P}$ = 8 µm, ${R}_{B}=$ 1 µm, ${T}_{B}=$ 14 µm, ${T}_{AlN}=$ 4 µm. ${P}_{P}$ = 3 ${L}_{P}$ while in (**b**) ${P}_{P}=$ 100 ${L}_{P}$ and the ambient is SiO_2_.

Figure 3a depicts the normalized far-field radiant intensity distribution averaged over the polar angle for ${L}_{P}=$ 8 µm and ${P}_{P}$=24 µm for each epi stack case, including the dissection of each case’s loss and extraction percentage. All three epi stack cases have similar far-field radiant intensity peaks at polar angles of approximately 40^∘^–60^∘^. Such radiant intensity profiles which exhibit a higher amount of power being sent to oblique angles are inherently tied to the considered DUV MQWs which also strongly emit oblique light in the epi, and the fact that the SVEP architecture outcouples more of such obliquely emitted light.

Figure 3b depicts the portion of absorption losses in 3 major grouped components of interest: the “P-side” (which includes the p-epi layers and the p-contact), the “N-contact”, and the “Al mirror”. “Extracted” reflects the percentage of power that reaches the SiO_2_ buffer region. The N-contact and Al buffer mirrors barely contributed to any losses, indicating that to improve the LEE further with the SVEP architecture, one should concentrate on reducing the p-side losses.

**Figure 4:**
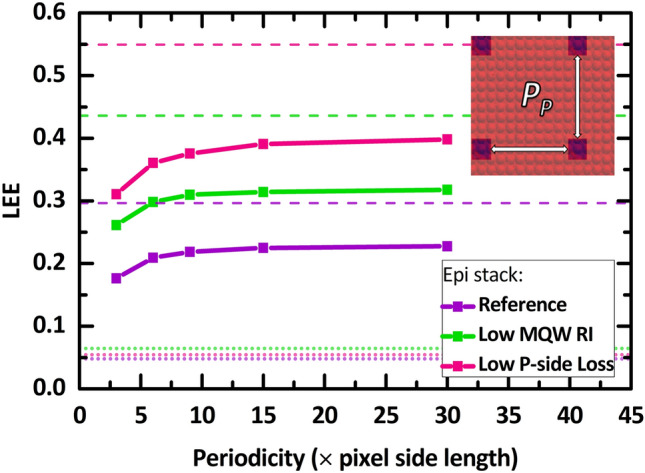
The LEE as a function of the pixel periodicity, ${P}_{P}$ as depicted by the inset. For this approach, the simulation was initialized with ${L}_{P}$ = 8 µm, ${R}_{B}=$ 1 µm, ${T}_{B}=$ 14 µm, and ${T}_{AlN}=$ 4 µm.

Figure 4 depicts the LEE as a function of the pixel periodicity, ${P}_{P}$ (visualized in the inset where each pixel is the blue square). Here, we consider a buffer layer texture radius ${R}_{B}$ of 1 µm, thickness ${T}_{B}$ of 14 µm, AlN thickness ${T}_{AlN}$ of 3 µm, and pixel side length ${L}_{P}$ of 8 µm. Increasing the pixel placement periodicity improves light extraction by increasing the probability of light rays reaching the top structured buffer/air interface prior to encountering neighboring pixels during their propagation. For all epi stack cases, the LEE increases drastically only at small ${P}_{P}$ magnitudes and reaches saturation points at higher magnitudes of ${P}_{P}$. Specifically, 3 ${L}_{P}$ to 6 ${L}_{P}$ of ${P}_{P}$ increase. The increase in LEE with increasing period is more prominent for the “Low P-side Loss” epi stack case, as this case provides the largest portion of power entering the buffer region. The increase in LEE is shown to saturate by ${P}_{P}=15{L}_{P}$ for all epi stack cases.

Comparing the SVEP architecture to the typical 1 $\times$ 1 mm^2^ sapphire chip, as highlighted by the small dotted lines, ${P}_{P}=3{L}_{P}$ the “Reference” case is $\sim$ 4$\times$, the “Low MQW RI” case is $\sim$ 4$\times$, and the “Low P-side Loss” is $\sim$ 5$\times$ greater than that of their sapphire chip counterparts. At the saturation points (${P}_{P}=15{L}_{P}$), one would achieve $\sim$ 6$\times$ of LEE improvement for all three cases. Considering both variations in the pixel side length and periodicity, a small pixel size relative to the pixel periodicity translates to a chip with small p-epilayers and an MQW, which increases the extraction through the epilayer sidewalls and reduces the losses in those major absorbing materials. The periodicity influencing the LEE is also affected by the placement of the pixels. Here, we consider a simple square lattice; other placement strategies may lead to significantly different results.

We note that a significant difference exists between the saturated LEE values at ${P}_{P}=15{L}_{P}$ and the maximum LEE values of each epi stack case. This difference stems from the imperfect outcoupling of light from the epi region into the buffer region due to the TIR at the epi/buffer, which causes light to return to the extremely absorbing p-epilayers. To a certain degree, one can expect that this problem could be alleviated by considering pixel shapes of higher symmetry such that there are more surfaces with different escape cone orientations. In addition, one could further increase the in coupling of light into the buffer region by having surface roughness at the epi edges or having a buffer layer with a higher index material but still not absorbing the emitted light.

### AlN layer thickness

**Figure 5:**
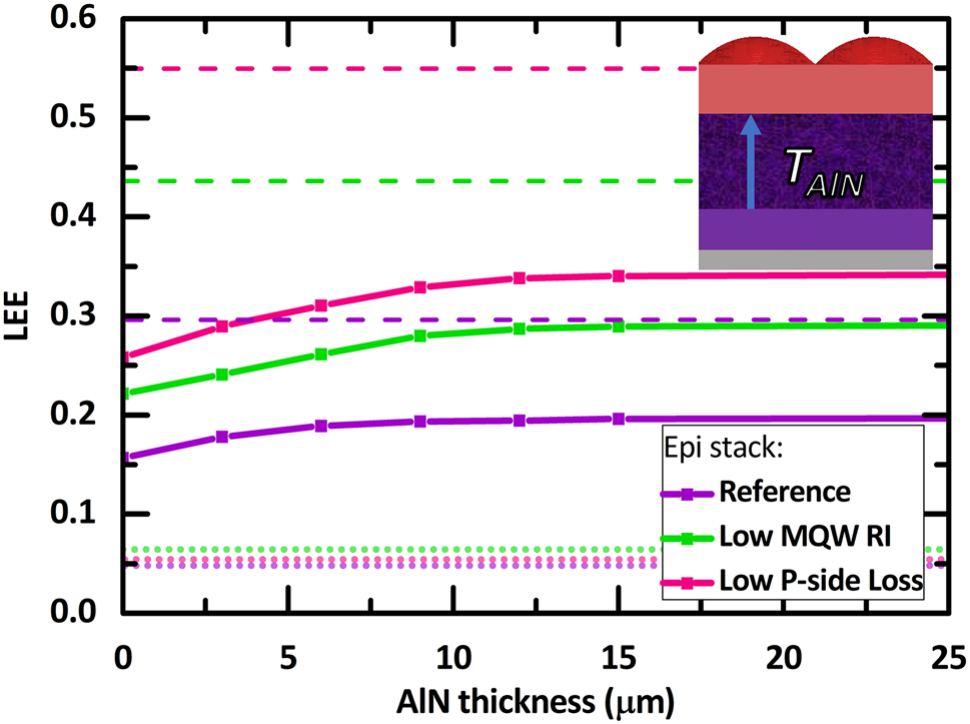
The LEE as a function of the aluminum nitride additional thickness. The inset is the visualization of the ${T}_{AlN}$ in a single pixel. The simulation was initialized with ${P}_{P}=$ 3 ${L}_{P}$, ${L}_{P}$ = 8 µm, ${R}_{B}=$ 1 µm, and ${T}_{B}$ is always 10 µm more than ${T}_{AlN}$.

Figure 5 shows the LEE performance with respect to AlN thickness, ${T}_{AlN}$. In these calculations, the buffer thickness is considered to always be 10 µm greater than that of AlN. Additionally, the other parameters remain constant at ${P}_{P}=$ 3 ${L}_{P}$, ${L}_{P}$ = 8 µm, ${R}_{B}=$ 1 µm, and ${T}_{B}=$ 10 $+{T}_{AlN}$. As depicted, there is a significant LEE improvement with increasing AlN thickness for each epilayer case, but the “Reference” epi stack case exhibits only up to $\sim 0.03$ an increase in the absolute LEE value. The “Low MQW RI” and “Low P-side Loss” epi stacks, on the other hand, show an absolute LEE value increase of up to ~ 0.06.

The LEE improvement is mostly due to the increased probability of light rays escaping through the sidewalls of the AlN layer before it returns to the lossy p-side, which naturally occurs as the thickness of that layer increases. We note that the AlN layer may have a significantly lower refractive index than the n-epi layer. In such cases, the TIR at the AlN/n-epi interface can cause a significant portion of the light to return to the lossy P-side region. At the optimum AlN thickness of ${T}_{AlN}=15$ µm, the “Low P-side Loss” epi stack case exhibits an LEE that is $\sim$ 5$\times$ larger than that pertaining to the large 1 × 1 mm^2^ sapphire chip architecture. The “Low MQW RI” and “Reference” epi stack cases exhibit an LEE that is $\sim$ 4$\times$ larger than that of their sapphire chip counterparts.

### Top buffer-nanotexture size

**Figure 6:**
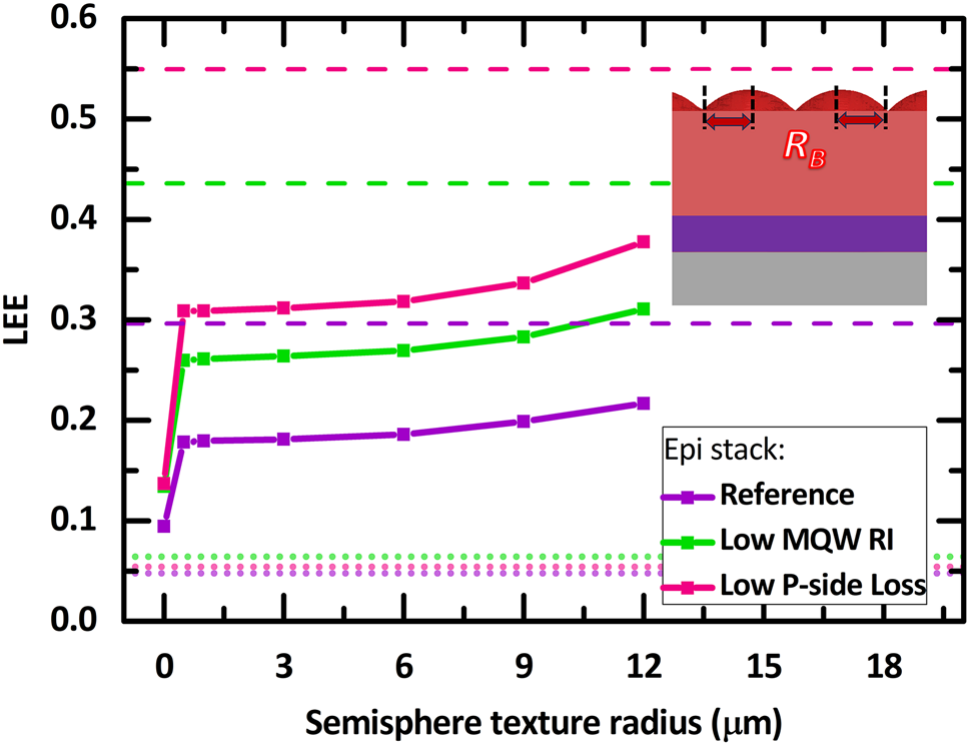
(**a**) The LEE as a function of the hemispherical-shaped, buffer sheet’s top-surface texture radius, ${R}_{B}$ that is depicted in (**b**) the single pixel side view. The constant parameters are ${P}_{P}=$ 3 ${L}_{P}$, ${L}_{P}$ = 8 µm, ${T}_{AlN}=$ 4 µm, and ${T}_{B}=$ 14 µm.

Figure 6 provides the LEE as a function of the hemispherical buffer texture radius. For our proof of principle purposes in the manuscript, we consider the buffer sheet’s top-surface texture to be densely packed micro-sized hemispheres, as depicted in the inset of Figs. 1 and 6. Here, the other relevant geometrical parameters are set to ${L}_{P}$ = 8 µm, ${T}_{AlN}$ = 4 µm, and ${T}_{B}=$ 14 µm for all points in Fig. 6. The LEE for all cases increases steadily as ${R}_{B}$ increases from 0.5 to 6 µm. Compared to the cases with a small texture radius, the LEE improvement is significant at a larger texture radius (more than 6 µm) for each epi case. This is because introducing hemispherical textures and increasing ${R}_{B}$ provides two factors for LEE improvement: providing multiple surface angles and reducing cross-coupling between neighboring textures.

Nevertheless, providing multiple surface angles is more significant than reducing texture cross-coupling. This can be deduced when the texture is introduced (even at ${R}_{B}=$ 0.5 µm), as the rays that encounter this hemispherical surface have multiple possible incident angles compared to those of a smooth planar surface (${R}_{B}=$ 0). Thus, the LEE drastically increased to 0.18 from 0.09 (higher magnitudes for the other two cases) as the TIR effect is broken. A significantly large monotonous increase of the LEE as ${R}_{B}$ increases from 6 to 12 µm is evident all epilayer cases. This dependence is attributed to the number and size of the texture features on top of a single pixel. Having less and bigger textures on top of a pixel reduces the probability of rays reflected by the top texture from reentering the pixel where it is emitted from.

At ${R}_{B}=$ 12 µm and a constant periodicity of ${P}_{P}=$ 3 ${L}_{P}= 24$ μm, there is only one complete hemispherical texture on top of a pixel. Increasing the texture radius by more than 3 ${L}_{P}$ would create overlaps between neighboring hemispheres within a pixel cell period, rendering them to be incomplete or imperfect hemispherical shapes and thus not considered. Note that we are considering these microsized hemisphere textures at the buffer/air interface only ray-optically (in the absence of wave-optical scattering effects). With diffraction scattering coming into play, the smaller texture sizes may actually produce a significantly larger LEE.

## Methodology

### Optical models and light source properties

The optical simulation is based on the combination of wave and ray optics models. The dipole emission of light into the n-epi is treated with wave optics following Wasey et al.’s analytical formulation of spontaneous dipole emission in a planar multilayer stack where the dipole radiation field is expended in terms of plane waves and the impact of the planar multilayer environment on the dipole emission characteristics is deduced from the transfer matrix response of each plane wave in that system^51^. Though we consider a more complex layer stack, our system can also be classified into 3 regions as considered by Wasey et al. The effective MQW layer is considered as region 1, the n-epi layer is considered as region 3 and the p-side substrate can be considered as region 2. One simply needs to adjust the transmission and reflection coefficients for the layer stack we consider, instead of the simple Fresnel reflection transmission coefficient of a single interface. Details on the equation used can be found in section 4 in the Supplementary.

We are thus approximating that the dipole radiation would not be affected by the sidewalls. Such an approximation would apply in cases where the pixels are large or if there is minimal light returning from the sidewalls due to outcoupling and absorption in the epilayers. The results of these calculations, namely, the portion of light that enters the n-epilayers, ${P}_{n}/{P}_{MQW}$ and its angular distribution, serve as input for our ray optical calculations. According to a recent paper, the orientations of the dipoles in the MQWs of typical UV-C LEDs emitting at approximately 265 nm with Al60Ga40N are potentially ~ 30% out of plane and 70% in plane^52^. For simplicity, we thus consider that the dipole emission orientation will on average be isotropic for all three epi stack variations^53^.

**Figure 7:**
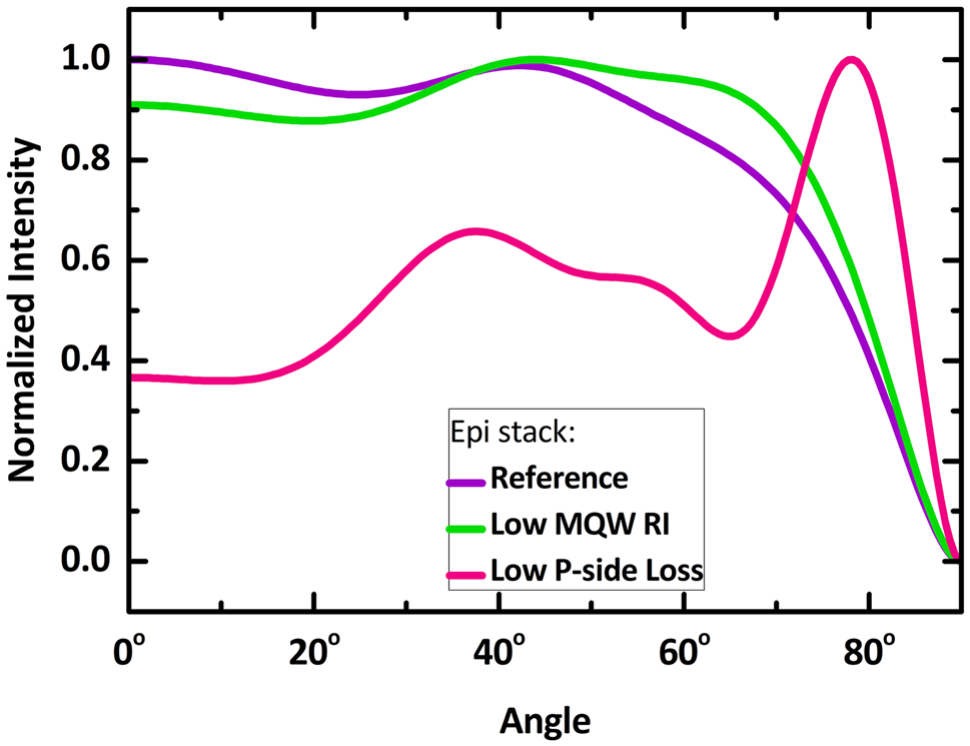
The radiant intensity of light emitted by the MQW into the n-epi as a function of emission angle for the considered three epilayer stack cases. Each is normalized to the maximum value. 0^∘^ represents the top direction while 90^∘^ is parallel to the lateral surface.

The ray optical portion of our modelling approach is essential in simulating the LEE performance of our structures, which are very large compared to the wavelength still. The ray tracing was performed utilizing LightTools software from Synopsys. The 3D pixel geometrical model is built in SolidWorks-3D. The launched normalized radiant intensity distributions in the n-epilayers for the three different epi stack cases are shown in Fig. 7. We consider a simplified monochromatic emission at a wavelength of 265 nm, as performed by López-Fraguas et al.^37^. The “Low P-side Loss” epi stack case exhibits a considerably stronger peak close to 80° that is not observed in the other two cases. This is inherently because light at such oblique angles is not absorbed as strongly in this epi stack case and has a greater possibility of escaping to the n-epilayers than in the other two epi stack cases.

The light tracing tool tracks both bulk absorption and surface losses. When light rays encounter most interfaces during the ray tracing, we simply consider Fresnel refraction laws. An exception is taken when light rays return from the n-side to the p-side of a pixel. There, we consider the reflection response of a multilayer planar stack which comprise of the whole p-epi layers and p-contact, in an attempt to consider the wave-optical effect in the absorption as well. To evaluate ray-tracing top extraction, $LEE$, a far-field hemispherical receiver was placed capturing the light propagated power from 0 ^∘^–90^∘^ elevation with

1 $$LEE=\frac{{P}_{n}-{P}_{Loss}}{{P}_{MQW}}.$$

where ${P}_{n}$ is the optical power reaching the n-epilayers, and ${P}_{MQW}$ is the effective source’s optical power. As the LEE numbers for each epi stack case is normalized relative to its own ${P}_{MQW}$ values, we ignore the differences of Purcell enhancement between stack and focus squarely on the extraction efficiency. ${P}_{Loss}$ is the total light loss incurred due to material absorption as light rays propagate in the structure. For further information on the LightTools simulation parameters and samples, please refer to the Supplementary third and fourth section.

## Conclusion

In conclusion, a novel scattered volume emitter micropixel architecture for DUV LEDs is presented. At the expense of an enlarged emission area with respect to the actual MQW active area, our Scattered Volume Emitter Pixels (SVEP) architecture allows one to achieve ultrahigh light extraction efficiency for DUV without the need for a nanoscale light outcoupling structure, a high-index substrate nor a lens. Simulations were performed to demonstrate the performance of the architecture in outcoupling light via a combined wave and ray optics approach. Three different epi stack flavors, which represent different cases of how much power enters the n-side of the epi layer, are considered. Based on our proposed architecture, the greater the amount of power that enters the n layers is, the greater the amount of light power that can be outcoupled. Even with a relatively large pixel size of 20 µm^2^ and a final emission surface area that is only 9 times larger than the MQW active area, the SVEP architecture can provide a light extraction efficiency that is 3–5× better than that of planar 1 $\times$ 1 mm^2^ LEDs on top of thick sapphire substrates, reaching up to > 30% LEE depending on the epi stack absorption. An even larger enhancement reaching 6$\times$ was also demonstrated with a pixel size of 8 $\times$ 8 µm^2^. Indeed, the trends show that even larger enhancements could be obtained with smaller pixel sizes and larger buffer textures, which are unfortunately not accessible with our current simulation approach due to our partial reliance on ray-optics. The SVEP architecture opens the possibility for DUV LEDs to rival the performance of much larger mercury lamps all while maintaining a significantly smaller total size.

### Supplementary Information

Supplementary Information.

## Data Availability

The datasets generated during and/or analyzed during the current study are available from the corresponding author upon reasonable request.

## References

[1] Gerchman Y, Mamane H, Friedman N, Mandelboim M (2021). Corrigendum to “UV-LED disinfection of Coronavirus: wavelength effect” [Journal of Photochemistry and Photobiology B: Biology 212 (2020) 112044–end page]. J. Photochem. Photobiol. B.

[2] Hsu T-C (2021). Perspectives on UVC LED: Its progress and application. Photonics.

[3] Wang C-P (2021). Efficiency improvement of batch reactors for water sterilization using UV-C LED arrays. Environ. Technol..

[4] Suzuki A, Emoto A, Shirai A, Nagamatsu K (2022). Ultraviolet light-emitting diode (UV-LED) sterilization of citrus bacterial canker disease targeted for effective decontamination of
* Citrus sudachi
* fruit. Biocontrol Sci..

[5] Jacob J, Pona A, Cline A, Feldman S (2020). Home UV phototherapy. Dermatol. Clin..

[6] Novoa RH, Huaman K, Caballero P (2022). Light-emitting diode (LED) phototherapy versus non-LED phototherapy devices for hyperbilirubinemia in neonates: A systematic review and meta-analysis. Am. J. Perinatol..

[7] Lazzarin M (2021). LEDs make it resilient: Effects on plant growth and defense. Trends Plant Sci..

[8] Ferreyra MLF, Serra P, Casati P (2021). Recent advances on the roles of flavonoids as plant protective molecules after UV and high light exposure. Physiol. Plant.

[9] Matsumoto T, Tatsuno I, Hasegawa T (2019). Instantaneous water purification by deep ultraviolet light in water waveguide:
* Escherichia coli
* bacteria disinfection. Water.

[10] Soro AB (2023). Current challenges in the application of the UV-LED technology for food decontamination. Trends Food Sci. Technol..

[11] Zou W, Sastry M, Gooding JJ, Ramanathan R, Bansal V (2020). Recent advances and a roadmap to wearable UV sensor technologies. Adv. Mater. Technol..

[12] Wang H (2020). Visible light activated excellent NO_2_ sensing based on 2D/2D ZnO/g-C_3_N_4_ heterojunction composites. Sens. Actuators B Chem..

[13] Kneissl M, Seong T-Y, Han J, Amano H (2019). The emergence and prospects of deep-ultraviolet light-emitting diode technologies. Nat. Photonics.

[14] Ren Z (2020). Band engineering of III-nitride-based deep-ultraviolet light-emitting diodes: A review. J. Phys. D Appl. Phys..

[15] Mondal RK, Adhikari S, Chatterjee V, Pal S (2021). Recent advances and challenges in AlGaN-based ultra-violet light emitting diode technologies. Mater. Res. Bull..

[16] Buonanno M, Welch D, Shuryak I, Brenner DJ (2020). Far-UVC light (222 nm) efficiently and safely inactivates airborne human coronaviruses. Sci. Rep..

[17] Glaab J (2021). Skin tolerant inactivation of multiresistant pathogens using far-UVC LEDs. Sci. Rep..

[18] Takano T (2017). Deep-ultraviolet light-emitting diodes with external quantum efficiency higher than 20% at 275 nm achieved by improving light-extraction efficiency. Appl. Phys. Exp..

[19] UV-C LED Product Specifications SMD 3535 Packaged LED 1. https://bolb.co/wp-content/uploads/2021/08/Bolb_SMD_3535_DR100_UVCLED-SpecSheet-V4.0.pdf.

[20] Zhao P, Han L, McGoogan MR, Zhao H (2012). Analysis of TM mode light extraction efficiency enhancement for deep ultraviolet AlGaN quantum wells light-emitting diodes with III-nitride micro-domes. Opt. Mater. Express.

[21] Yue Q, Li K, Kong F, Zhao J, Liu M (2016). Analysis on the effect of amorphous photonic crystals on light extraction efficiency enhancement for GaN-based thin-film-flip-chip light-emitting diodes. Opt. Commun..

[22] Yun J, Hirayama H (2017). Investigation of the light-extraction efficiency in 280 nm AlGaN-based light-emitting diodes having a highly transparent p-AlGaN layer. J. Appl. Phys..

[23] Chen Q (2019). Enhanced optical performance of AlGaN-based deep ultraviolet light-emitting diodes by electrode patterns design. IEEE Electron Device Lett..

[24] Zhang S (2019). Enhanced wall-plug efficiency in AlGaN-based deep-ultraviolet LED via a novel honeycomb hole-shaped structure. IEEE Trans. Electron Devices.

[25] Nagasawa Y, Hirano A (2019). Review of encapsulation materials for AlGaN-based deep-ultraviolet light-emitting diodes. Photonics Res..

[26] Ye ZT (2019). Nanoparticle-doped polydimethylsiloxane fluid enhances the optical performance of AlGaN-based deep-ultraviolet light-emitting diodes. Nanoscale Res. Lett..

[27] Kang C-Y (2019). A novel liquid packaging structure of deep-ultraviolet light-emitting diodes to enhance the light-extraction efficiency. Crystals.

[28] Zhang L (2019). Deep ultraviolet light-emitting diodes based on a well-ordered AlGaN nanorod array. Photonics Res..

[29] Park JY (2020). Subwavelength-scale nanorods implemented hexagonal pyramids structure as efficient light-extraction in light-emitting diodes. Sci. Rep..

[30] Cho HK (2017). Highly reflective p-contacts made of Pd-Al on deep ultraviolet light-emitting diodes. IEEE Photonics Technol. Lett..

[31] Kashima Y (2018). High external quantum efficiency (10%) AlGaN-based deep-ultraviolet light-emitting diodes achieved by using highly reflective photonic crystal on p-AlGaN contact layer. Appl. Phys. Express.

[32] Cho HK (2020). Enhanced wall plug efficiency of AlGaN-based deep-UV LEDs using Mo/Al as p-contact. IEEE Photonics Technol. Lett..

[33] Khizar M, Fan ZY, Kim KH, Lin JY, Jiang HX (2005). Nitride deep-ultraviolet light-emitting diodes with microlens array. Appl. Phys. Lett..

[34] Zhou L (2006). Vertical injection thin-film AlGaN∕AlGaN multiple-quantum-well deep ultraviolet light-emitting diodes. Appl. Phys. Lett..

[35] Lee D (2017). Improved performance of AlGaN-based deep ultraviolet light-emitting diodes with nano-patterned AlN/sapphire substrates. Appl. Phys. Lett..

[36] Zheng Y (2019). Effects of meshed p-type contact structure on the light extraction effect for deep ultraviolet flip-chip light-emitting diodes. Nanoscale Res. Lett..

[37] López-Fraguas E (2022). Tripling the light extraction efficiency of a deep ultraviolet LED using a nanostructured p-contact. Sci. Rep..

[38] Yan J (2022). A vertical AlGaN DUV light-emitting diode fabricated by wafer bonding and sapphire thinning technology. Appl. Phys. Express.

[39] Muramoto Y, Kimura M, Nouda S (2014). Development and future of ultraviolet light-emitting diodes: UV-LED will replace the UV lamp. Semicond. Sci. Technol..

[40] Kawasaki K, Koike C, Aoyagi Y, Takeuchi M (2006). Vertical AlGaN deep ultraviolet light emitting diode emitting at 322nm fabricated by the laser lift-off technique. Appl. Phys. Lett..

[41] Aoshima H (2012). Laser lift-off of AlN/sapphire for UV light-emitting diodes. Physica Status Solidi C.

[42] Cho HK (2017). Chip design for thin-film deep ultraviolet LEDs fabricated by laser lift-off of the sapphire substrate. Semicond. Sci. Technol..

[43] Fujii K (2009). Leakage current improvement of nitride-based light emitting diodes using CrN buffer layer and its vertical type application by chemical lift-off process. Appl. Phys. Lett..

[44] Cho C-Y (2011). Growth and separation of high quality GaN epilayer from sapphire substrate by lateral epitaxial overgrowth and wet chemical etching. Appl. Phys. Express.

[45] Picardi MF, Manjavacas A, Zayats AV, Rodríguez-Fortuño FJ (2017). Unidirectional evanescent-wave coupling from circularly polarized electric and magnetic dipoles: An angular spectrum approach. Phys. Rev. B.

[46] Getty ARK, David A, Wu Y, Weisbuch C, Speck JS (2007). Demonstration of distributed Bragg reflectors for deep ultraviolet applications. Jpn. J. Appl. Phys. Part 2 Lett..

[47] Alhenc-Gelas, C. *et al.* Design rules of high reflectivity Bragg GaAlN mirrors for 300nm VCSELs. In *Proc.SPIE* Vol. 7229, 72290N (2009).

[48] Zheng T (2024). Refractive index engineering as a new degree of freedom for designing high-performance AlGaN-based ultraviolet C light-emitting diodes. Adv. Photonics Res..

[49] Zheng T (2023). In-depth insights into polarization-dependent light extraction mechanisms of AlGaN-based deep ultraviolet light-emitting diodes. Opt. Express.

[50] Liu C, Melanson B, Zhang J (2020). Algan-delta-gan quantum well for DUV LEDs. Photonics.

[51] Wasey JAE, Safonov A, Samuel IDW, Barnes WL (2000). Effects of dipole orientation and birefringence on the optical emission from thin films. Opt. Commun..

[52] Finn R, Schulz S (2022). Impact of random alloy fluctuations on the electronic and optical properties of (Al, Ga)N quantum wells: Insights from tight-binding calculations. J. Chem. Phys..

[53] Römer F (2024). Carrier transport in a deep ultraviolet mixed quantum well light emitting diode. IEEE Photonics J..

