# Supplementary material for: A scattered volume emitter micropixel architecture for ultra efficient light extraction from DUV LEDs
Source: Sci Rep. 2024 Jun 19;14:14108. doi: 10.1038/s41598-024-64689-y (PMC11187205; doi:10.1038/s41598-024-64689-y)
Supplement: Supplementary file 1 — Supplementary Information. [file 41598_2024_64689_MOESM1_ESM.pdf]

**SUPPLEMENTARY INFORMATION FOR**

# A scattered volume emitter micropixel architecture for ultra efficient light extraction from DUV LEDs

**Faris Azim Ahmad Fajri[1,2,*], Anjan Mukherjee[1], Suraj Naskar[1], Ahmad Fakhrurrazi Ahmad Noorden[2], and Aimi Abass[1]**

[1] ams OSRAM Group, Leibnizstraße 2, Regensburg, Germany.

[2] Centre for Advanced Optoelectronics Research, Kulliyyah of Science, International Islamic University Malaysia, Kuantan Pahang, Malaysia.

[*]faris-azim.ahmad-fajri@ams-osram.com

## Validation of the optical simulation

Our combined ray and wave optical simulations in this work are validated by modeling the LEE of the planar reference considered by López-Fraguas et al. [42]. The epilayer properties are tabulated in Table S1, where the only difference from the properties of the SVEP epilayers (as discussed in the main text) is the thick sapphire block on top. Note that the n-side losses are omitted due to the transparency (or significantly low extinction) of the 265 nm emission.

| Materials | | Thickness | Refractive index |
|---|---|---|---|
| 1 | Pt | 0.40 µm | 1.1653 + i2.4307 |
| 2 | GaN | 40 nm | 2.6981 + i0.476 |
| 3 | AlGaN | 75 nm | 2.6103 + i0.340 |
| 4 | AlGaN | 25 nm | 2.4774 + i0.119 |
| 5 | MQW | 18 nm | 2.9532 |
| 6 | AlGaN | 1.00 µm | 2.3723 |
| 7 | AlN | 4.00 µm | 2.3147 |
| 8 | Sapphire | 0.40 mm | 1.8337 |

**Table S1.** Epilayer thickness and optical properties of a "Reference" epi stack case.

We reproduced an LEE of 4.6% for the case of a semi-infinite LED stack with a sapphire superstrate considering an in-plane dipole emission only from multiple quantum wells (MQW). Instead, as shown in Fig. S1, considering the finite 1×1 $mm^2$ size of the chip and allowing rays to escape from the sidewalls and assuming an isotropic dipole orientation in the MQW, as explained in the main text, we obtained an LEE of 4.8%. Note that rays that exit the chip in the downward direction are also considered in the LEE calculation, as we assume these rays can be used.

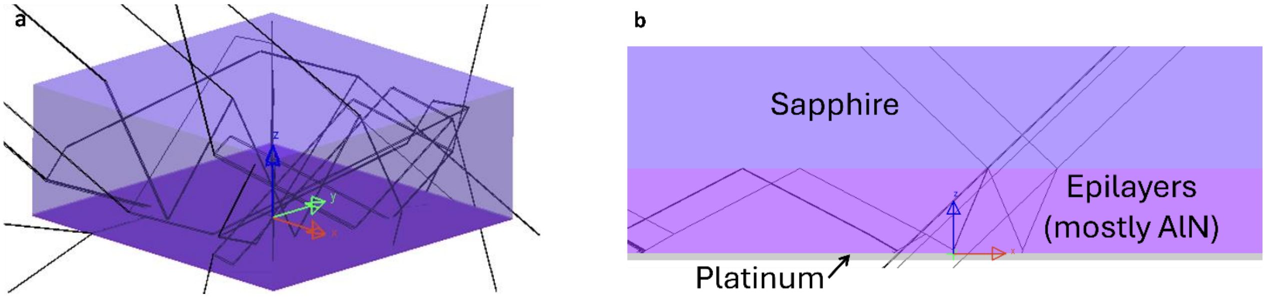


**Figure S1**. The 3-dimensional raytracing of 10 rays for the planar 1×1 $mm^2$ sapphire chip. (**a**) is the overall 3-dimensional view which is mostly sapphire block, and (**b**) is the magnified 2-dimensional XZ view where the thick, blue-colored block is sapphire, the purple block which is mostly AlN is the epilayers, and the thin gray layer is platinum.

The hybrid ray and wave optics LEEs for this simplified 1×1 $mm^2$ chip architecture for the three considered epistack cases are summarized in Table S2. As the referenced epilayer case is validated, the other two epilayer stack cases (as discussed in the main text) were chosen to further analyze the extraction capability of the SVEP architecture, i.e., a stack with a lower MQW refractive index and another stack with a lower p-side loss. For completeness, we include simulated LEE of the 1×1 $mm^2$ chip without the sapphire substrate on top. In a reference 1x1mm^2 chip, the main exit surface of light from the semiconductor epitaxial layers is only the top surface as not a lot of light emitted in the middle region of the chip will reach the epi sidewalls. With a thick sapphire substrate on top, one allows a major portion of the light which lies within the escape cone of epi to sapphire to be extracted and subsequently be outcoupled into air at the sapphire top and sidewall interfaces. The sapphire essentially act as a volume emitter. Without the sapphire, light will be confined within the lossy semiconductor DUV epi and a major portion of it will be lost.

| Epilayer stack | LEE (%) by Combined Ray and Wave optics |
|---|---|
| Reference | 4.8 |
| Reference without sapphire substrate | 2.9 |
| Low MQW RI | 6.5 |
| Low MQW RI without sapphire substrate | 3.8 |
| Low P-side Loss | 5.4 |
| Low P-side Loss without sapphire substrate | 3.0 |

**Table S2.** Simulation results of LEE based on our combined ray and wave optical modeling.

Although the "Low P-side Loss" epistack case has relatively less absorption than the "Reference" and "Low MQW RI" cases, its LEE is quite low. The main reason is that a large amount of power is sent to oblique angles in the n-epi at ~70 degrees beyond the escape cone of the n-epi to the sapphire. Because we only consider a planar chip structure, light beyond the n-epi/sapphire escape cone remains trapped in the semiconductor epilayers. The SVEP architecture exploits this very same condition by considering small micropixels architecture where light can also escape from the sidewalls of the epi into the buffer layer region.

## Geometric variations with negligible LEE enhancement

Table S3 summarizes what is studied for Figs. 2-6 in the main text and other parameters that are kept constant for each figure. The parameters and study set are to mainly highlight physics instead of providing an optimum structure.

| Design Parameter | | Variation Range | Remarks |
|---|---|---|---|
| Figure 2 | Pixel side length, $L_P$ | 8.0 – 160.0 (μm) | $T_B$, $R_B$, and $T_{AlN}$ are 14 μm, 1 μm, and 4 μm, respectively. |
| Figure 4 | Pixel periodicity, $P_P$ | 3.0 – 30.0 (× $L_P$ ) | $L_P$, $T_B$, $R_B$, and $T_{AlN}$, are 8 μm, 14 μm, 1 μm, and 4 μm, respectively. |
| Figure 5 | AlN thickness, $T_{AlN}$, | 0.1 – 30.0 (μm) | $T_B = (T_{AlN} + 10$ μm). $L_P$, $P_P$, and $R_B$ are 8 μm, 3$L_P$, and 1 μm, respectively. |
| Figure 6 | Buffer sheet nanotexture radius, $R_B$ | 0.5 – 12.0 (μm) | $L_P$, $T_B$, $P_P$, and $T_{AlN}$ are 8 μm, 14 μm, 3$L_P$, and 4 μm, respectively. |

**Table S3**. The chip design variation approaches.

Supplementary

## Buffer thickness

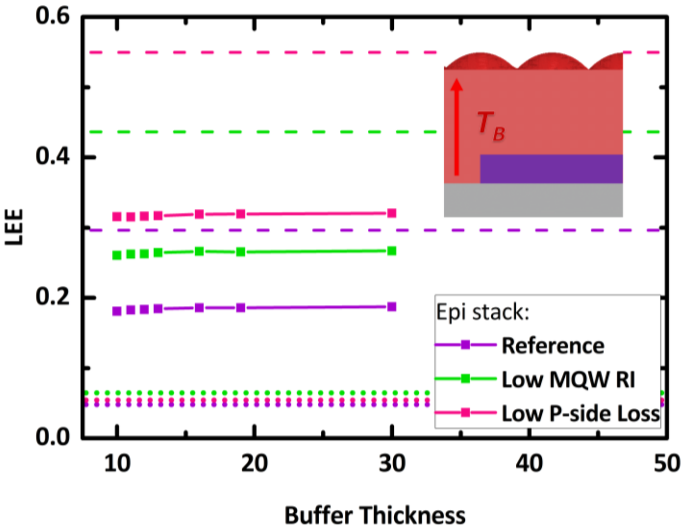


**Figure S2**. The LEE as a function of buffer thickness variation. Here, $L_P$ = 8 µm, $R_B$ = 1 µm, $T_B$ = 14 µm, $T_{AlN}$ = 4 µm, and $P_P = 3L_P$.

Varying the buffer thickness, as depicted in Fig. S2, has a trivial effect on the LEE performance. This is true for all epistack cases. However, compared to their 1×1 mm$^2$ sapphire counterparts, our SVEP architecture outperforms the LEE by at least 3 folds. This is because the increased thickness is actually beneficial for sidewall extraction, but due to the large emission area compared to the MQW size, most of the rays are extracted upward rather than through the buffer sidewalls. Therefore, the effect of varying the buffer thickness alone on the LEE is negligible.

## Buffer sheet air volume and percentage.

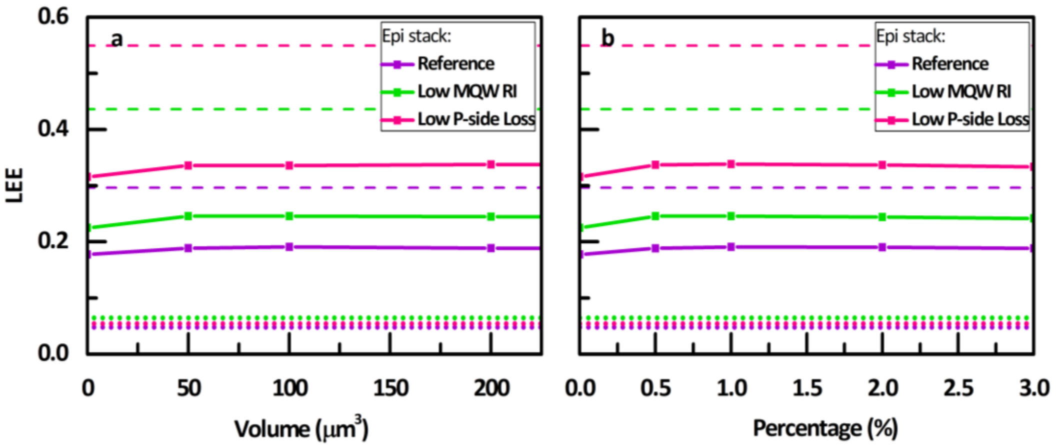


**Figure S3**. The LEE performance by the buffer sheet's air volume and percentage variations. Here, $L_P$ = 8 µm, $R_B$ = 1 µm, $T_B$ = 14 µm, $T_{AlN}$ = 4 µm, and $P_P = 3L_P$.

In Fig. S3, similar to the buffer thickness variation, the a) air volume and b) percentage variations of the buffer sheet do not have a major effect on the LEE. Nonetheless, compared to a buffer sheet without volume scatterers (the volume and percentage of air particles are zero), when air particles are in the buffer sheet, there is a slight increase in the LEE by $\sim$0.02 for all epilayer cases. This means that scattering slightly improves the extraction in this buffer medium, but increasing the scattering probability does not further increase the extraction efficiency. While the scattering increases the variety of rays' incident angles on the extraction surface, it also increases the TIR probability, which counteracts the efficiency by redirecting the rays into the absorbing layers.

## Sample of data and results

The LEE ray-tracing calculations were performed with a minimum of 100,000 rays for each case of geometric and material epilayer property variations. We found that the LEE values converged with such a number of rays. The far-field shown in Figure 3 of the main paper was computed with 5 million rays. Fig. S4 shows an example of the SVEP's single-pixel ray tracing simulation with a 100-ray setting, where the gray block is platinum, and the red block is the $SiO_2$ buffer layer covering that single pixel.

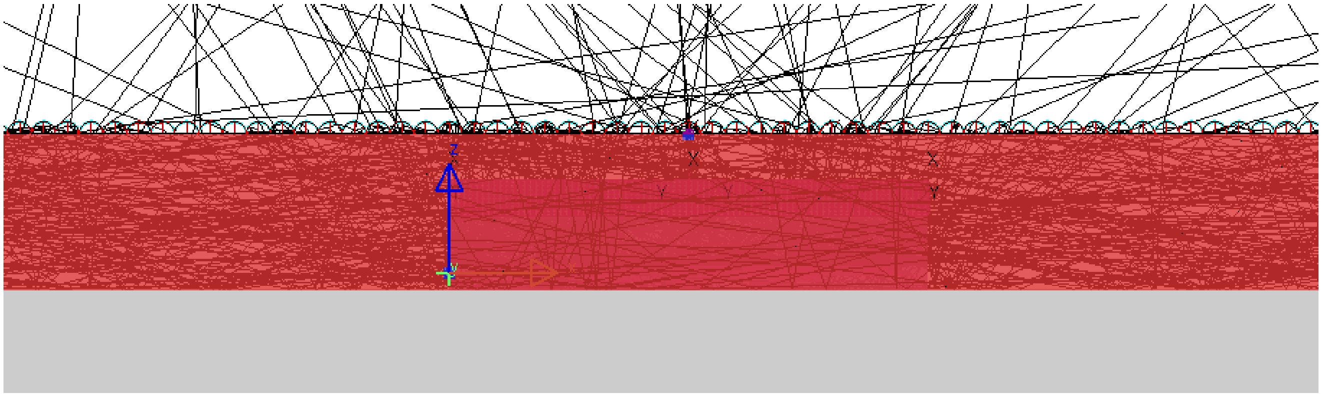

.

**Figure S4**. A sample of SVEP ray tracing simulation with a sample of 100 rays. This is a YZ-axis 2-dimensional view. The gray-colored block is platinum, and the red block is the $SiO_2$ buffer sheet covering the single pixel.

Notably, most variations of complex geometrical parameters require us to utilize design-based software, Solidworks-3D, in which the complete model can be exported into Lighttools for ray-tracing simulation afterward. For example, when the pixel size is varied, the changes in complex curves, filleting, and layers' subtraction can only be done in design-based softwarewithout error. As noted in the main text, the light tracing tool tracks both bulk absorption and surface losses, and also collects light that escapes the structure and propagate into the far-field in the ambient media (in our case we consider air as ambient except in Fig. 2(c) ). When light rays encounter most interfaces during the ray tracing, we simply consider Fresnel refraction laws. An exception is taken when light rays return from the n-side to the p-side of a pixel. There we consider the reflection response of a multilayer planar stack which comprise of the whole p-epi layers and p-contact, in an attempt to consider the wave-optical effect in the absorption as well. The more round trips occur in the absorbing layers due to Fresnel reflection and TIR, the more absorption takes place. We currently ignore photon recycling (reabsorption and reemission) processes in the MQWs as we consider at operational conditions the MQWs would be heavily pumped and be naturally less absorbing. In the considered epi stacks which exhibit large p-side losses, the effect of photon recycling would be overshadowed by the absorption losses in the surrounding material. Throughout the manuscript and supplementary the ray-tracing settings have been described, but for simplicity, they are arranged accordingly in Table S4.

| Simulation parameters | Reference | Low MQW RI | Low P-side Loss |
|---|---|---|---|
| Light source power ratio | 0.2964 | 0.4361 | 0.5496 |
| Light source radial intensity | As in Figure 7 | | |
| Light source spectrum | 265 nm | | |
| Light receiver angular coverage | 0°-90° | | |
| Ambient material | Air | | |
| Material optical properites | As in Table 1 | | |
| Minimum number of rays | More than 100,000 | | |
| Minimum power threshold | $1\times10^{-5}$ | | |

**Table S4.** Ray-tracing parameters.

## Additional details on wave optics formulation

The total radiated power portion $P_n$ is inferred by integrating the radiant intensity that goes to the n-epi layer[52].

$$P_n = \frac{q}{4}\int_0^{\frac{\pi}{2}} F(u)\sin\theta_n \, d\theta_n \tag{s1}$$

where $u = k_n \sin\theta_n$ is the in-plane wavevector, $k_n$ refers to the wave-vector in the n-epi material, with $u^2 = \sqrt{k^2 + l^2}$ as $l$ correspond to the wave vector normal to the plane. $q$ is the quantum efficiency of the emitting dipole (assumed to be 1 as we only consider LEE in this contribution). The subscript $n$ simply refers to the n-epi layer after the MQW. The radiation intensity, $F(u)$ into the n-epi layers is calculated according to eq. (16) in their paper[52] but adjusted for our considered layer stacks. For completeness, we restate it here,

$$F(u) = \frac{k_n l_n^2}{k_{MQW}^3}\left(k_{MQW}^2 \frac{\varepsilon_{MQW}}{\varepsilon_n}\left|\frac{T_{\parallel\tilde{s}\uparrow}}{l_{MQW}} exp\left(il_{MQW}t\right)\right|^2 + \left|T_{\parallel\tilde{s}\uparrow} exp\left(il_{MQW}t\right)\right|^2 + u^2\left|\frac{T_{\perp\tilde{p}\uparrow}}{l_{MQW}} exp\left(il_{MQW}t\right)\right|^2\right) \tag{s2}$$

The subscript *MQW* Refers to the effective multiple quantum well layer. $T$ represents the composite transmission coefficient of the stack, ∥ and ⊥ denote the dipole's horizontal and vertical orientation, respectively, while $\tilde{s}$ and $\tilde{p}$ signify the polarization of the plane wave components. Thus, with these parameters, the LEE is normalized based on the source optical power, $P_{MQW}$

$$\begin{aligned} P_{MQW} = (1-q) &+ \frac{q}{2k_{MQW}^3}\int_0^\infty u^2\left(\left(1 + R_{\perp\tilde{p}\uparrow} exp(v)\right) + \left(1 + R_{\perp\tilde{p}\downarrow} exp(w)\right)\right)\frac{u}{l_{MQW}} du \\ &+ \frac{q}{2k_{MQW}^3}\int_0^\infty k_{MQW}^2\left(\left(1 + R_{\parallel\tilde{s}\uparrow} exp(v)\right) + \left(1 + R_{\parallel\tilde{s}\downarrow} exp(w)\right)\right)\frac{u}{l_{MQW}} du \\ &+ \frac{q}{2k_{MQW}^3}\int_0^\infty l_{MQW}^2\left(\left(1 + R_{\parallel\tilde{p}\uparrow} exp(v)\right) + \left(1 + R_{\parallel\tilde{p}\downarrow} exp(w)\right)\right)\frac{u}{l_{MQW}} du \end{aligned} \tag{s3}$$

Here, $v = 2il_{MQW}b$ and $w = 2il_{MQW}t$, where $b$ refers to the distance from the point dipole to the MQW/p-epilayer interface, while $t$ is equivalent to $b$ but with respect to the MQW/n-epilayer. Note that $T$ and $R$ comprises of other transmission and reflection coefficients from the MQW layer to the p-side and n-side. Their expression are given in the appendix of Wasey et al.'s paper [52] equivalent as the following

$$\begin{aligned} R_{\parallel,\perp\uparrow} &= \frac{r_{MQW\leftrightarrow p}\left(r_{MQW\leftrightarrow n}e^{w} \pm 1\right)}{1 - r_{MQW\leftrightarrow p} r_{MQW\leftrightarrow n} e^{v+w}} \\ R_{\parallel,\perp\downarrow} &= \frac{r_{MQW\leftrightarrow n}\left(r_{MQW\leftrightarrow p}e^{v} \pm 1\right)}{1 - r_{MQW\leftrightarrow p} r_{MQW\leftrightarrow n} e^{v+w}} \\ T_{\parallel,\perp\uparrow} &= \frac{t_{MQW\leftrightarrow p}\left(1 \pm t_{MQW\leftrightarrow n}e^{w}\right)}{1 - t_{MQW\leftrightarrow p} t_{MQW\leftrightarrow n} e^{v+w}} \\ T_{\parallel,\perp\downarrow} &= \frac{t_{MQW\leftrightarrow n}\left(1 \pm t_{MQW\leftrightarrow p}e^{v}\right)}{1 - t_{MQW\leftrightarrow p} t_{MQW\leftrightarrow n} e^{v+w}} \end{aligned} \tag{s4}$$

For these expressions, they are used in the same manner for both $\tilde{s}$ and $\tilde{p}$ plane wave components where $r$ and $t$ here refer to the reflection and transmission coefficients of the corresponding polarization. They are normalized based on the full emissive layer's components i.e., with length, $d = b + t$.
